# Glioma Stem Cells: Novel Data Obtained by Single-Cell Sequencing

**DOI:** 10.3390/ijms232214224

**Published:** 2022-11-17

**Authors:** Alisa Gisina, Irina Kholodenko, Yan Kim, Maxim Abakumov, Alexey Lupatov, Konstantin Yarygin

**Affiliations:** 1 Laboratory of Cell Biology, V.N. Orekhovich Institute of Biomedical Chemistry, 119121 Moscow, Russia; 2 Drug Delivery Systems Laboratory, D. Mendeleev University of Chemical Technology of Russia, 125047 Moscow, Russia

**Keywords:** intratumoral heterogeneity, glioma stem cells, astrocytoma, oligodendroglioma, glioblastoma, adult-type diffuse gliomas, high-throughput single-cell analysis, single-cell sequencing, single-cell omics, CAR-T cells

## Abstract

Glioma is the most common type of primary CNS tumor, composed of cells that resemble normal glial cells. Recent genetic studies have provided insight into the inter-tumoral heterogeneity of gliomas, resulting in the updated 2021 WHO classification of gliomas. Thorough understanding of inter-tumoral heterogeneity has already improved the prognosis and treatment outcomes of some types of gliomas. Currently, the challenge for researchers is to study the intratumoral cell heterogeneity of newly defined glioma subtypes. Cancer stem cells (CSCs) present in gliomas and many other tumors are an example of intratumoral heterogeneity of great importance. In this review, we discuss the modern concept of glioma stem cells and recent single-cell sequencing-driven progress in the research of intratumoral glioma cell heterogeneity. The particular emphasis was placed on the recently revealed variations of the cell composition of the subtypes of the adult-type diffuse gliomas, including astrocytoma, oligodendroglioma and glioblastoma. The novel data explain the inconsistencies in earlier glioma stem cell research and also provide insight into the development of more effective targeted therapy and the cell-based immunotherapy of gliomas. Separate sections are devoted to the description of single-cell sequencing approach and its role in the development of cell-based immunotherapies for glioma.

## 1. Introduction

Historically, gliomas were classified based on anatomical location and histopathological pattern. The degree of malignancy (increasing from grade I to grade IV) was determined by the extent of cellular atypia and mitotic activity [1]. In the 2007 WHO classification, glial tumors were arranged into groups according to their morphology, and the term “glioblastoma” denoted astrocytic, oligodendroglial or oligoastrocytic gliomas with microvascular proliferation and necrosis [2]. Later, data on genetic alterations in glioma cells were used to explain the differences in the course of the disease in patients with identical diagnoses. As a result, in the 2016 classification of CNS tumors, molecular features in addition to histology were used to define tumor entities [3]. In 2021, the WHO updated the classification of CNS tumors in accordance with the latest data concerning the key genetic mutations and the DNA methylation status [4]. Specifically, astrocytomas, oligodendrogliomas and glioblastomas are currently regarded as different tumor entities combined into the group of “adult-type diffuse gliomas”. Notably, glioblastomas have the wild type, nonmutated IDH (isocitrate dehydrogenase) gene, while astrocytomas are IDH-mutant, and oligodendrogliomas are IDH-mutant and also have the 1p/19q codeletion (see Table 1) [5]. Besides providing insight into the inter-tumoral heterogeneity, detailed genetic analysis of gliomas delivers important prognostic information that allows prediction of the disease course at the diagnosis stage and guides the choice of the optimum treatment options [6,7,8,9,10,11,12].

Along with optimizing the treatment protocols according to the genetic status, the development and clinical trials of targeted drugs and various types of immunotherapy for gliomas are underway [13,14,15,16]. However, for a large number of patients, the effectiveness of the applied treatment still remains low. For example, the 5-year relative survival rate for glioblastoma patients according to the latest data is only about 7% [17]. Thus, new breakthrough approaches in this field of research are needed to develop really effective methods of treatment. Studies of the intratumoral glioma cell heterogeneity at the single-cell level are rapidly gaining momentum and look quite promising with regard to the advancement of more effective knowledge-based therapies [18].

The actuality of cancer stem cells (CSCs) is one of the manifestations of the intratumoral heterogeneity. These cells exhibit some phenotypic and functional properties of normal stem cells of the same tissue. It has been hypothesized that CSCs are responsible for tumor formation and progression [19,20]. In gliomas, a CSC subpopulation has also been identified and its tumor-initiating properties, as well as the ability for asymmetric division, have been documented [21,22,23]. Unfortunately, no consensus on the markers of glioma stem cells was found because the results of different studies were controversial. Moreover, there is evidence of genetic heterogeneity in the subpopulation of glioblastoma cells expressing common glioma stem cell markers [24]. Thus, it is not clear whether CSCs represent a separate tumor population or whether they are just a manifestation of a certain phenotypic state that many cancer cells can assume under certain conditions. The development of the single-cell omics approach has made it possible to overcome the traditional limitations and solve experimental problems requiring single-cell resolution.

Here we review the recent results obtained by the single-cell expression profiling of glioma stem cells. A separate section is devoted to discussing the interrelation of single cell sequencing with other methods of high-throughput single-cell analysis and the possibility of combining them in the intratumoral heterogeneity research. Moreover, we outline the prospects of immunotherapy based on the single-cell sequencing of gliomas.

## 2. Intratumoral Heterogeneity and the Current Concept of Cancer Stem Cells

Malignant tumors, including glioma, comprise different cell types, such as cancer cells, stromal cells, endothelial cells and immune cells [25]. In addition, cancer cells actively proliferate, accumulate mutations and form genetically different clones contributing to the enhancement of tumor cell diversity [26]. Moreover, under the influence of stimuli coming from the microenvironment, epigenetic changes occur in individual cancer cells [27,28,29]. All this produces a vast heterogeneity of tumor cells, specific for each patient and even for each site of tumor growth [30,31,32]. This is an important practical aspect, since it causes the difference in the clinical manifestation of cancer in different patients. Obviously, the prognosis of the disease progression depends on the presence or absence of drug-resistant or metastasizing tumor cells [33,34,35,36].

**Figure 1:**
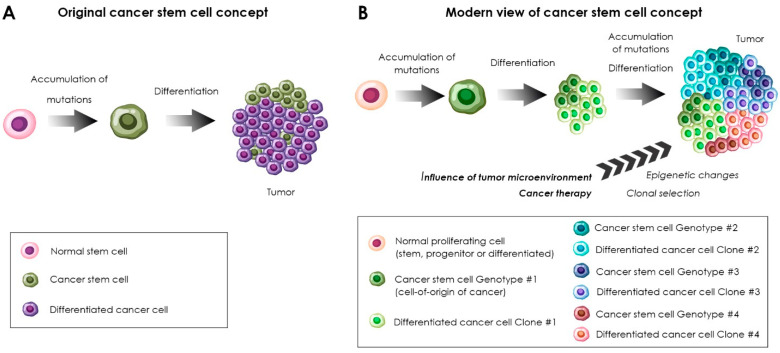
Evolution of the cancer stem cell concept. The original concept (**A**) emerged on the basis of the stem cell theory in its original form [19] and on the reports about the exclusive ability of tumor cells expressing stem cell markers to induce tumor growth [38,43]. The modern concept (**B**) has been updated by introducing the concept of the cells-of-origin of tumors [58,61] and according to data obtained by genetic analysis combined with CSC-associated marker profiling [24] and lineage tracing analysis [59,60,62,63]. Notably, the relationship between the cells-of-origin of cancer and the CSCs is yet not well understood and the characteristics of both cell types may dynamically change. The process of the CSC differentiation involves the activation of the rare quiescent stem cell-like subpopulation which gives rise to the progenitor-like actively proliferating cells and the subsequent generation of non-stem cell-like, non-cycling, non-tumorigenic “differentiated” cancer cells.

In experiments using cell-sorting technologies (flow cytometry, magnetic separation) in combination with the in vivo cell transplantation to the immunodeficient animals, it was found that cells isolated from the same tumor differ by their tumor-initiating capacity [37,38,39,40,41,42,43,44,45,46,47]. The most tumorigenic were subpopulations of tumor cells expressing the markers of normal resident stem cells. The term “cancer stem cells” came into use to designate this kind of cell. Alternatively, some researchers use the term “cancer stem-like cells”. The presence of cancer stem cells (CSCs) was shown initially in hematological [37,39], and later in solid malignancies [20,21], including glioma [48,49,50,51,52,53]. According to the hierarchical theory of oncogenesis, the tumor originates from a mutated stem cell and has a hierarchical cell composition [19,38]. Cancer stem cells are at the top of the hierarchy, and all other tumor cells are their differentiated progeny (see Figure 1A). Over time, evidence supporting the existence of a subpopulation of cancer cells with stem cell features has accumulated. However, the hierarchical theory did not find determinate confirmation and has been reconsidered [54]. It is likely that tumors combine the following features: genetic mosaicism manifested by the presence of clones with the ability to invade, metastasize and/or resist to drugs, epigenetic changes in cancer cells under the influence of the tumor microenvironment and the existence of subpopulations within separate subclones that have functional and molecular similarities to stem cells [54,55]. Moreover, one should distinguish the terms “cancer stem cell” and the “cell-of-origin” [56]. The proper definition for the cell-of-origin of cancer is “cancer-initiating cell”. Cancer stem cells, on the other hand, are the cells that maintain tumor propagation. According to the current views, different transformed cells, including stem cells, progenitors or even differentiated cells may be implicated as the cells-of-origin in tumor initiation in different cancers [57,58]. Experiments using genetic barcoding in vivo [59] and lineage tracing by means of single-cell RNA sequencing analysis [60] showed that the stem-like subpopulation of cancer cells consists of rare quiescent cells and a large population of frequently proliferating cells which give rise to non-cycling cells without a “stem-like signature”. For example, in a study of the clonal evolution of barcoded glioblastoma cells during serial xenotransplantation [59], it was demonstrated that slowly proliferating stem-like cells give rise to more frequently dividing progenitor cells with an extensive self-maintenance capacity, which proceed to the formation of non-proliferative cells. The authors also found rare “outlier” clones that deviated from this proliferative hierarchy and showed that chemotherapy enhances the expansion of pre-existing drug-resistant glioblastoma stem cells [59]. The scheme demonstrating the contemporary view of the cancer stem cell concept is shown in Figure 1B.

## 3. Molecular Markers and Functional Features of Glioma Stem Cells

Many transcription factors or structural proteins required for the functioning of normal stem and progenitor cells, including Sox2, Nanog, Olig2, Myc, Musashi1, Bmi1, Nestin, Oct4, Brn2 and inhibitor of differentiation 1 (ID1) are present in glioma stem cells Table 2). The surface cell markers of glioma stem cells include CD133, CD15 (also referred to as SSEA-1), α6 integrin, CD44, L1CAM, CD24, EGFR, PDGFRA and A2B5 (Table 2) [62,64,65,66,67,68,69,70,71,72,73,74,75]. The surface marker-based approach is convenient for enriching the pool of CSCs from the tumor bulk using flow cytometry or magnetic separation. However, each particular marker is not necessarily expressed by all glioma stem cells. This conclusion was drawn from the studies which showed that cells not expressing a particular glioma stem cell marker can be tumorigenic in vivo and, moreover, reconstitute the initial tumor heterogeneity and give rise to cells expressing this glioma stem cell marker [76,77,78]. Therefore, functional tests demonstrating stem cell properties are required to provide more accurate evidence confirming that a cell actually belongs to the CSC subpopulation. The functional features of the CSCs include tumor formation after xenotransplantation in the immunodeficient animals and the ability to form spheroids during in vitro culturing [41,48]. It is important to note that isolated cells placed in a new microenvironment can change their state, no matter what approach is used to enrich them. In particular, cell culturing induces changes in the expression of surface molecules and alters the biological state of glioma cells [79]. The fact that each tumor, apparently, contains different glioma stem cells belonging to genetically different subclones [24] is probably the key factor of the controversy concerning glioma stem cell markers (see Figure 1B).

In this regard, a detailed study of the phenotypic and functional heterogeneity of glioblastoma stem cells without a mutation in the IDH1 gene, carried out by Galdieri et al. using mass cytometry, is of great interest [80]. This paper assessed the relevance of CD15, CD44, CD133, and α6 integrin, which earlier proved to be convenient for identifying tumor-initiating glioblastoma cells, as glioblastoma stem cell surface markers. Using freshly isolated tumor samples from patients with glioblastoma, as well as cell cultures derived from them, the authors studied how CSC populations identified by each single marker correlate with regard to intracellular signaling and cell function, and whether the expression of various combinations of these markers is associated with differences in the tumor-forming ability. The authors measured the expression of intracellular markers of normal stem cells that are involved in the proliferation, migration, and tumorigenesis, such as Sox2, Musashi, Nanog, and Nestin, as well as the activation of key developmental signaling pathways that are routinely activated in glioblastoma stem cells, such as PI3K/AKT, MEK/ERK, JAK/STAT, WNT/β-catenin, NF-κB and MAPK/P38. As a result, 15 different subpopulations of glioblastoma stem cells were found in tumor samples, and they differed in the status of activation of the MEK/ERK, WNT and AKT signaling pathways. The authors found that during culturing some subpopulations were lost, and previously undetected subpopulations appeared. Glioblastoma cells, which strongly expressed all four surface markers of glioma stem cells (CD15^high^ CD44^high^ CD133^high^ α6 integrin^high^ phenotype), had the highest self-renewal capacity, sensitivity to the WNT inhibitor and tumorigenicity in vivo. According to this study, WNT/β-catenin and NF-κB were the main signaling pathways associated with the glioblastoma stem cells. Intracellular proteins associated with normal stem cells were expressed in both CSCs and non-CSCs [80].

Due to the diversity of glioma stem cell subtypes, high-throughput single-cell analyses is the most suitable approach to assess cellular heterogeneity and to identify CSC subpopulations in gliomas.

## 4. Single-Cell Sequencing Approach

In the intratumoral cell heterogeneity research the most abundant and essential information is currently provided by single-cell sequencing [81]. This technology combines many different methods that allow studies of the transcriptome [82,83,84], genome [85], methylome [86] or other chromatin modifications [87] in single cells. The number of analyzed cells from one sample ranges from hundreds to tens of thousands. The number of measured parameters for each cell reaches several thousands. Thus, single-cell sequencing provides big data, the analysis of which requires specialized bioinformatic tools.

### 4.1. Separation of Individual Cells

In the genetic analysis of single cells, an important step in the sample preparation is the process of the isolation and labeling of each individual cell. Here, dissociation is the key step carried out through mechanical mincing, enzyme treatment and shaking. To prevent excessive cell lysis in order to avoid damage to genetic material, it is necessary to select the optimal dissociation technique for each type of tissue [88]. Isolation of individual cells from the cell suspension can be performed employing the limiting dilution approach, micromanipulation using microscopy, fluorescence-activated cell sorting (FACS) or laser capture microdissection [89]. The limiting dilution approach is a simple and inexpensive method for isolating single cells [90]. However, to avoid multiple cells in one well, the dilution is made to be excessive, and thus some of the wells for sample preparation remain empty. To obtain extracts from early embryos or non-culturable microorganisms, micromanipulation under the control of microscopy is the classic method [91,92] while FACS is used to isolate single cells with specific characteristics [93]. The disadvantage of this technology is the traumatization of the cell membrane during the sorting process, which can lead to apoptosis and RNA damage [94]. In addition, FACS requires a large initial number of cells (at least thousands). Alternatively, cells for analysis can be isolated from fixed tissue sections by laser capture microdissection [95].

Microfluidic technologies represent novel methods of single-cell isolation and labeling, which are now increasingly implemented into practice [96,97]. In one widely used microfluidic technology, the isolation of single cells is achieved by the sequential intersection of the cell suspension stream with a stream of special gel microparticles and a hydrophobic medium (oil) stream [98,99]. This process results in the formation of an oil drop containing a gel particle with a cell attached to it. The microparticle is made of sepharose or agarose and is covered with single-stranded oligonucleotides comprising a universal sequence for attaching an adapter for sequencing, a unique sequence of a gel particle for labeling RNA from one cell, unique for each oligonucleotide UMI (unique molecular identifier) for labeling each individual RNA molecule and a polythymidine tail to capture the poly(A) tail of the eukaryotic mRNA. The cells are suspended in a buffer containing the reaction mixture for cell lysis, RNA extraction and cDNA synthesis. Another successful technology for single-cell isolation and labeling is based on the cell “capture” in microwells [100]. This technology employs special cartridges of microwells fitting just one cell and one unique magnetic microsphere. Each magnetic microsphere is coated with single-stranded oligonucleotides with a structure similar to that described above. After a cell and a magnetic microsphere fit in the well, the cell is lysed and its RNA content is captured by the oligonucleotides on the surface of the magnetic microsphere. Confinement and flushing of the magnetic particles in the cartridge wells are controlled by a magnet installed into the bottom of device [100].

### 4.2. Preparation of Genetic Material and Sequencing

In the case of single-cell RNA sequencing, the further protocol consists of the following steps: reverse transcription of isolated RNA into first-strand cDNA, cDNA amplification, cDNA library preparation, sequencing and bioinformatic analysis of the obtained data [101]. In the case of single-cell DNA sequencing, the reverse transcription step is not required [102]. After cell isolation and DNA extraction, the amplification procedure is carried out. The method of the whole genome amplification varies depending on whether the detection of large regions is needed to analyze copy number variation (CNV), or relatively short sequences are deciphered to spot, for example, single nucleotide variations (SNV) or indels (insertion/deletion) [103].

Most single-cell sequencing methods are based on second-generation sequencing technologies, which provide a simultaneous reading of many small cDNA fragments ranging from several tens to several hundreds of nucleotides [104,105]. The cDNA library construction is a laborious multi-step process that includes cDNA fragmentation, adapter ligation and selection of cDNA fragments of desired length. Meanwhile, third-generation sequencing technologies are rapidly coming into practice. Their important advantage is the direct sequencing of DNA or RNA molecules without previous reverse transcription and amplification [106,107]. Among the third-generation sequencing solutions there are two long-read sequencing technologies—the Pacific Biosciences (PacBio) single-molecule real-time (SMRT) sequencing [108] and the Oxford Nanopore Technologies (ONT) nanopore sequencing [109]—through which it is possible to read a continuous nucleic acid sequence of more than 20 thousand nucleotides in real time. Long-read sequencing provides the opportunity to determine the presence of nucleic acid modifications in single molecules at base resolution and without specialized chemicals that can be damaging to DNA [110]. Long reads allow the phasing of base modifications with genetic variants, enabling the research of epigenetic heterogeneity [111]. Furthermore, long-read sequencing enables the analysis of base modifications in repetitive regions of the genome (centromeres or transposons), where short reads cannot be mapped uniquely [110,112]. It is worth noting though, that both SMRT and nanopore technologies provide a lower per read accuracy than short-read sequencing [110]. In the case of SMRT, the quality of sequencing is an inverse function of the length of the original fragment and the polymerase longevity. In case of nanopores, the quality of reads is independent of the length of the DNA fragment but depends on achieving the optimal translocation speed of the nucleic acid through the pore, which typically decreases in the late stages of sequencing runs, negatively affecting the quality [110]. At the present time, approaches combining short and long read sequencing technologies are being developed [110,113]. In terms of the long read sequencing application to single-cell analysis, nanopore sequencing is currently in higher demand [112,114,115]. 

### 4.3. Bioinformatic Analysis of Single-Cell Sequencing Data

The algorithm for the bioinformatic analysis of single-cell sequencing data includes the evaluation of the sequencing quality parameters, removal of adapters and low quality sequences, alignment of reads to the reference version of the human genome, filtering of PCR duplicates, detection of variants that differ from the reference genome and their filtering according to quality [116]. Sequencing results are then uploaded to the online databases, such as the Gene Expression Omnibus (GEO) or The Cancer Genome Atlas (TCGA) [117], become publicly available and are actually used by other researchers. For example, Pang et al. [118] utilized single-cell RNA-seq data from the study of Patel et al. [119] to discover rare genes associated with a minor subset of glioblastoma cells. Pang et al. found that these rare genes tended to be specifically expressed in glioblastoma stem cells and also were enriched in a cell subset, which exhibited high cell cycle activity and invasive potential [118].

Since highly expressed genes produce a lot of RNA, more cDNA is produced during its reverse transcription and more cDNA sequences are read during sequencing compared to genes that are less expressed. Therefore, RNA sequencing is a method for the quantitative analysis of gene expression, because the number of reads of cDNA sequences is correlated with the expression level of a particular gene in a cell [120].

Using bioinformatic tools, single-cell sequencing data can be used to investigate cell heterogeneity. To accomplish this, dimensionality reduction techniques (PCA, t-SNE, UMAP) and clustering are used, followed by the analysis of the identified cell subtypes [120]. As a result, bioinformatic dissection of single-cell RNA sequencing data allows a model of tissue cell composition to be developed. In the case of cancer research, cells, after primary quality control and data normalization, are classified into widely different cell types (malignant, endothelial and others) based on the expression of a set of marker genes and identified genomic aberrations. At the next stage, within each of these cell types, subpopulations are identified, each corresponding to a different cell state and with a unique expression program. Thus, cell states (stem-like, progenitor, differentiated), developmental cell types, proliferating cells (cycling, non-cycling) and genotypes of these cell states (copy-number aberrations, mutations, rearrangements) are identified. The obtained data are compared among patients with the same tumor type in order to identify similar states and correlated with datasets from other studies to clarify their biological significance [121].

To model the dynamic biological processes (cell cycle, cell activation, differentiation), the pseudo-temporal trajectory method is used [120]. The basic assumption of this method is that all single cells of the studied tissue are at different stages of a dynamic process. The pseudo-temporal trajectory has a linear form with bifurcations, similar to a tree trunk and branches. Trajectory modeling reveals progenitor cells or determines the stage at which a cell fate decision for differentiation is made. Many modern methods for constructing a pseudo-temporal trajectory require the introduction of additional information, for example, about marker genes or progenitor cells from which the trajectory will begin. However, this approach may be biased, depending on what additional information a particular researcher will use. Therefore, there are a number of tools that rely on prior knowledge of the studied object, and build a trajectory based solely on measured data (for example, Monocle DDRTree, Sincell and TSCAN) [120]. Recently a method, named Slingshot, which does not require any a priori knowledge about the lineages, was presented [122]. If there is no prior biological information, Slingshot can be applied in an unsupervised manner. If researchers already have some information about the cell types present in the samples, they can systematically integrate it [122]. The pseudo-temporal trajectory approach was successfully applied in the recently published study of the tumorigenic trajectory from human neural stem cells to malignant glioma cells [123] and in the research of intermediate glial progenitors and cell-fate decisions during the genesis of gliomas [124].

### 4.4. Single-Cell Multimodal Omics Approach

Single-cell sequencing makes it possible to detect cell populations, including small and sparse ones, and the transcriptional activation of signaling pathways, but it does not give the picture of translation of the genomic and transcriptomic variations into changes in the proteomic component. For the latter task, such high-throughput single-cell technologies as flow cytometry [125,126], imaging flow cytometry [127,128,129,130] and mass cytometry [131,132,133,134,135] are indispensable and can be combined with single-cell genetic analysis for research purposes (see Table 3). 

Innovative approaches that allow simultaneous genetic and proteomic analysis of single cells, for example using antibodies labeled with oligonucleotides containing unique barcode sequences, are now emerging [136]. Moreover, even more complex multimodal omics approaches combining the simultaneous profiling of epigenetic features, DNA sequences, gene expression, proteome, metabolic activity, etc., in single cells are being actively developed. However, to date such methods have a relatively limited throughput and a low coverage of the epigenome, transcriptome and other “omes” in individual cells [137].

## 5. Single-Cell RNA Sequencing in the Analysis of Glioma Stem Cell Subpopulations

Despite its short history, the single-cell RNA sequencing already provided enough data to construct a hypothetical model of the cell composition of different types of gliomas. It turned out that the cell composition differs in IDH-mutant gliomas and in gliomas carrying wild-type IDH. At the same time, the cell composition of different types of IDH-mutant gliomas is similar.

### 5.1. The IDH-Mutant Gliomas—Oligodendroglioma and Astrocytoma

**Figure 2:**
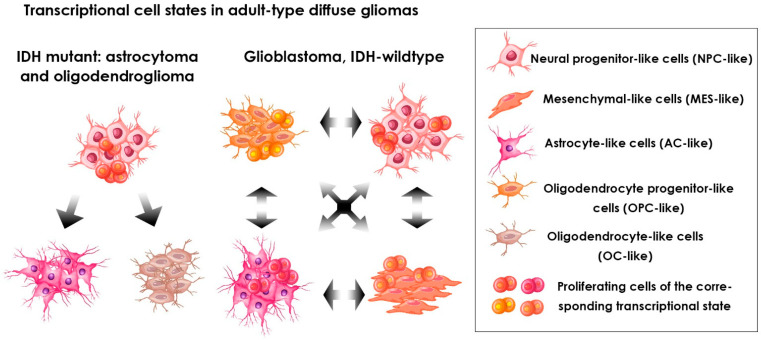
Transcriptional cell states in adult-type diffuse gliomas revealed by the single-cell RNA sequencing performed by Venteicher et al. [141] and Neftel et al. [142]. The cell states were defined based on the stemness/differentiation and proliferation signatures. Arrows represent revealed transitions between transcriptional cell states.

The IDH-mutant gliomas include oligodendroglioma and astrocytoma. These two glioma types differ in histology and genetics. Oligodendroglioma cells also have a chromosomal codeletion 1p/19q, while astrocytoma cells contain mutations in the TP53 and ATRX genes [138]. Previously, due to their morphology and differential staining with the astrocyte marker GFAP (glial fibrillary acidic protein) these gliomas were thought to originate from two major glial cell lineages, oligodendrocytes and astrocytes. However, recently Augustus et al. revealed the presence of largely nonoverlapping populations of SOX9+ astrocyte-like and OLIG1+ oligodendrocyte-like tumor cells in diffuse grade II IDH1-mutant gliomas, both oligodendrogliomas and astrocytomas [139]. Furthermore, single-cell RNA sequencing studies performed by Tirosh, Venteicher and their colleagues [140,141] showed that oligodendroglioma and astrocytoma share a similar cell hierarchy, including three major subpopulations: stem/progenitor-like cells, cells similar to neural progenitor cells (NPC-like cells) and two subpopulations of differentiated glia-like cells, similar to oligodendrocytes (OC-like) and astrocytes (AC-like). Importantly, proliferating cells are mainly the NPC-like cells [140,141] (see Figure 2). According to these data, the CSCs of the IDH-mutant gliomas proliferate more strongly than the more differentiated malignant cells. The authors hypothesized that the described cell composition limits the rate of tumor growth, and probably explains the slow spread of IDH-mutant gliomas. 

These data also conform with the standard hierarchical theory, with subpopulations of undifferentiated cells driving tumor growth. Involvement of the NPC-like tumor cells in the production of both the OC-like and the AC-like cells partly replicates the process of glial and neural differentiation during normal development. However, differentiation into neuron-like cells does not occur, being possibly blocked through molecular mechanisms dependent on the IDH1/2 mutations. It was also demonstrated that the high-grade lesions contained more undifferentiated and proliferating cells and showed higher infiltrating macrophages/resident microglial cells ratio compared to the low-grade lesions [141].

### 5.2. Glioblastomas, IDH-Wild Type

Gliomas with wild-type IDH are referred to as glioblastomas [5]. Verhaak et al. analyzed data on gene expression in glioblastomas cataloged in TCGA and found that glioblastomas may be classified into four molecular subtypes—proneural, neural, classical and mesenchymal [143]. Having in mind this classification, Steponaitis and Tamasauskas [144] performed comparative single-cell RNA sequencing analysis of human glioblastoma derived stem-like cells NCH421K and human glioblastoma cell line U87-MG using the nanopore sequencing system. The authors revealed branching of mesenchymal and proneural glioblastoma subtypes that was significantly linked to patient outcome and assumed the existence of distinct glioblastoma stem cell subpopulations. Furthermore, recent studies using single-cell RNA sequencing have demonstrated that each individual glioblastoma contains many subpopulations of cells that belong to different subtypes, and most tumors contain cells that are typical for at least three different subtypes [119,142,145]. These subpopulations differ significantly in their relative frequency in individual tumors and most glioblastomas predominantly contain one of the subtypes. According to data obtained by Neftel et al., a high level of EGFR amplification is associated with tumors characterized by a high frequency of astrocyte-like cells (AC-like), a high level of PDGFRA amplification occurs in tumors with high frequency of oligodendrocyte progenitor-like cells (OPC-like) and a high level of CDK4 amplification is predominant in tumors that contain a high percentage of neural progenitor-like (NPC-like) cells. Tumors containing large numbers of mesenchymal-like cells (MES-like) were characterized by changes in the NF1 gene, infiltration by immune cells and hypoxia [142]. Thus, the glioblastoma subtypes previously identified by Verhaak et al. [143] seem to reflect the preponderance of various cell states rather than their exclusive representation in the tumor. An emerging model of the glioblastoma cell composition suggests that this tumor includes four types of malignant cells: three of them share similarities with other classes of glioma and are closely related to cell types involved in the development of the nervous system (NPC-like, OPC-like and AC-like), and the fourth (MES-like) is not related to neural development [142]. Importantly, the proliferating cells in glioblastomas, unlike the described above IDH-mutant types in gliomas, were found among cells of each of the four cell states (see Figure 2). In addition, it has been demonstrated that when xenografts enriched with cells in a particular state were implanted in mice, the growing tumor recapitulated the initial ratio of cell states that was observed in the primary human tumor, suggesting the possible plasticity of these states. The identity of such “common” cell states can at least partly arise from tumor genetics, since specific genetic alterations only predispose cells remaining in certain states to proliferate, while other cell states are reduced to lower numbers [142]. Thus, each of these cell states can be interpreted as having a unique ability to self-reproduce and differentiate, and, accordingly, each state can be designated as glioblastoma stem cells, constituting the top of a unidirectional cell hierarchy. This may result in a situation when different groups of researchers isolate cells in different states under the same name “glioblastoma CSCs”, therefore obtaining contradictory results. For example, using single-cell RNA sequencing of the glioblastoma stem cells isolated from clinical tumor samples, Richards et al. demonstrated a transcriptional diversity of the glioblastoma stem cell fraction that could not be fully explained by DNA somatic alterations [146]. Two major transcriptional signatures of glioblastoma stem cells, namely the neurogenic and the mesenchymal/inflammatory, corresponded to their two major states [146].

To correlate the published evidence on the functionally defined glioblastoma stem cells with the single-cell RNA sequencing-based cell states described above, Tirosh and Suvà examined data on the expression of known markers of glioblastoma stem cells taken from a dataset generated using single-cell RNA sequencing [121]. The authors demonstrated that the level of CD24 was highest in the NPC-like cells, CD133 in the OPC-like cells, EGFR in the AC-like cells and CD44 in the MES-like cells. Among the transcription factors and lineage markers, Nestin showed a significant bias towards the AC-like cells. Other markers tested were expressed in two of the four states or lacked a recognizable pattern. This analysis based on mRNA levels was consistent with data from the FACS experiments employing labeled antibodies against the markers: CD24-positive cells were enriched with the NPC-like cells and CD44-positive cells were enriched with the MES-like cells [142]. Taken together, these results indicate that different markers of glioblastoma stem cells distinguish between different cell states and call for caution when interpreting traditional glioblastoma stem cell experiments.

Couturier et al. performed single-cell RNA sequencing on 53,586 adult glioblastoma cells and 22,637 normal human fetal brain cells and compared the lineage hierarchy of the developing human brain to that of cancer cells. The authors found four cancer cell lineages which closely resembled the signatures described by Neftel et al. [142]. Furthermore, at the intersection of these lineages Couturier et al. found the fifth cell type, with the progenitor cell-like transcriptome and functionally similar to the apical glioma stem cells [145]. The closest transcriptomic parallel of this cell cluster in the normal developing human brain was the glial progenitor cells. Both progenitor cell types, (GPCs) and OPCs, expressed PDGFRA and OLIG2, but Nestin expression was restricted to GPCs. Notably, the GPC signature was almost exclusively restricted to the CD133-positive sorted cells [145].

Similar findings were made in a recent study by Bhaduri et al., who performed single-cell RNA sequencing of glioblastoma cells and revealed that multiple glioblastoma stem cell subtypes exist within a single tumor [147]. The authors argued that the cellular composition of a tumor directly depends upon the subtype or subtypes of glioblastoma stem cells contained within this tumor. For example, the main cell type of glioblastoma stem cells in one of the studied tumor samples was dividing OPCs, and more than 30% of the cells constituting the tumor were oligodendrocytes. In other cases, the cellular composition of the tumor samples was more heterogeneous and matched several glioblastoma stem cell types. Bhaduri et al. identified an unusual outer radial glia-like cell population, similar to the cell type found during normal cortical development and capable of undergoing a characteristic mitotic somal translocation (MST), the so-called ‘‘jump-and-divide.’’ Since MST behavior enables the stem cell niche to expand during normal brain development, the authors suggested that in tumors these cells play a role in the expansion and invasion [147]. Concurrently, Wang et al. [148] demonstrated that adult human glioblastomas contain a subpopulation of radial glia-like cells displaying typical normal human fetal glial morphology and markers. This population included transcriptionally dynamic clusters of cells in the alternative quiescent and cycling states. The authors suggested that radial glia cells could represent the cells of origin or the CSCs residing at the top of the glioblastoma cell hierarchy [148]. Hamed et al. tried to obtain a better understanding of the association between the cerebral cancers cell types and the resembling stages of neural development [149]. They mapped the development of the mouse cerebrum, from the embryonic day 12.5 to the postnatal day 365, performing single-cell transcriptomics on >100,000 cells. Then the authors compared their reference atlas to the single-cell data from >100 glial tumors of the adult and pediatric human cerebrum. Primary data were obtained from the prior studies [141,142,146,150]. It was found that tumor cells have an expression signature overlapping with that of the embryonic radial glial precursors and their immediate sublineages [149].

Interesting data were obtained by Dirkse et al. [151], who showed that phenotypic heterogeneity based on cell membrane markers in glioblastoma cultures is a dynamic process of reversible state transitions. All glioblastoma subpopulations exhibited stem cell properties and were tumorigenic, indicating a high plasticity of cancer cells in response to microenvironmental influences. Despite the absence of a phenotypic hierarchy, the authors noticed functional differences. Specifically, differences in tumor development in vivo were linked to the time required by each subpopulation to reach the final environment-specific heterogeneity. The authors suggested that high tumorigenic potential is characteristic of cells that are able to quickly adapt and quickly restore the intratumoral phenotypic heterogeneity. The authors hypothesized that this may explain the inconsistencies demonstrated by different investigators in the functional studies in vivo and in vitro. These data indicate that in addition to the diversity of glioblastoma stem cells, the transcriptional plasticity also seems to exist. Cancer cells under the microenvironment influence can undergo reversible transitions between states enhancing their adaptive capacity. Furthermore, Auffinger et al. revealed the conversion of differentiated cancer cells into cancer stem-like cells in a glioblastoma model after primary chemotherapy [152]. The authors showed that the exposure of patient-derived glioma cells and also established glioma cell lines to temozolomide consistently increased the pool of glioblastoma stem cells over time both in vitro and in vivo. The newly converted glioblastoma stem cells expressed markers associated with pluripotency and stemness, such as CD133, SOX2, Oct4 and Nestin. An intracranial implantation of the newly converted glioblastoma stem cells in nude mice resulted in a more efficient grafting and invasive phenotype. The authors suggested that this interconversion between non-stem and stem-like states represents a potential mechanism for therapeutic relapse [152].

## 6. The Role of the Single-Cell RNA Sequencing in the Development of Cell-Based Immunotherapies for Glioma

Since gliomas do not respond well to conventional therapy, immunotherapy is considered a promising tool to fight this disease. Lymphocytes can cross the blood–brain barrier and attack cells bearing tumor-associated antigens (TAAs). Among the cell-based immunotherapeutic approaches, the therapy with genetically engineered chimeric antigen receptor (CAR) T cells seems to be the most developed. In addition to the recognition domain of a TAAs-specific antibody and CD3ζ signaling domain, the CAR-T cells of the latest generation usually include genetic modifications that increase the efficiency of their activation and resistance to apoptosis [153]. Since the CAR-T cells are designed to recognize surface antigens of tumor cells, they are not restricted by MHC and do not depend on the antigen processing. For this reason, routine flow cytometry of surgically dissected tumor tissue can be used to assess the expression of predefined TAAs and to determine the eligibility of the patient for the CAR-T cell therapy. Unfortunately, a number of preclinical and clinical trials using various TAAs as a target have shown only moderate benefits in the treatment of gliomas with CAR-T cells [15,154]. It is most likely that one of the main reasons for the lack of the CAR-T cells efficacy is the high inter-tumoral and intratumoral cell heterogeneity in gliomas. 

To counter the negative impact of the intratumoral cell heterogeneity, CAR-T cells should be directed to the top of the tumor cell hierarchy. For this reason, TAAs associated with the CSC phenotype including CD133 [155], EPH Receptor A2 (EphA2) [156], and L1CAM [157] can be used as a target for immunotherapy [158]. Apparently, incomplete characterization of the glioma stem cell phenotype of different glioma subtypes may be the reason for the poor clinical response to the CAR-T cells therapy. There is evidence that in the case of gliomas, CSC-like cells within the tumor bulk may belong to distinct subpopulations [159]. If so, the targeted cells can be replaced by another CSC subpopulation with similar properties, which implements the same or alternative signaling pathway, but differs in its surface antigens. Single-cell sequencing is a good choice for studying CSC diversity, because it can reveal stem cell-specific expression patterns without pre-separation of the cells based on their surface markers. To target distinct subpopulations of glioma stem cells that may simultaneously reside within one tumor, a new generation of CAR-T cells with multiple TAAs specificity is needed. For example, CAR-T cells simultaneously targeting IL13Ra2, HER2 and EphA2 were obtained by the co-transduction of three independent genetic constructs [160]. One more way to generate multispecific CAR -T cells is to endow them with the ability to secrete bispecific T cell engagers (BiTEs) with additional antigenic specificity. Such CART.BiTE cells were effective despite tumor heterogeneity in a mouse glioma model [161]. 

The tumor microenvironment also appears to be responsible for the failures of the CAR-T cell therapy of gliomas. There is evidence that the CSC niche is able not only to maintain the stem cell phenotype, but also to induce stemness in differentiated tumor cells [162]. In this case, the elimination of CSCs will have a transient effect and CSCs will be restored due to the CSC plasticity. Another important factor reducing CAR-T cells’ efficiency is an immunosuppressive microenvironment in glioma lesions. The presence of a large number of myeloid suppressors and the absence of tumor-infiltrating lymphocytes have been shown in the glioma microenvironment [163,164]. Thus, the outcome of cell-based immunotherapy can probably be improved by using not only tumor cells, but also the tumor microenvironment as its target. Single-cell RNA-sequencing is a very useful tool for identifying the precise therapeutic targets among cells in the tumor microenvironment. For example, expression analysis at the single-cell level of 201,986 cells isolated from human gliomas revealed myeloid cell subtypes with prognostic significance. In addition, the protein S100A4 was identified as a promising target, since its elimination leads to a change in the immunosuppressive landscape [165]. A similar single-cell RNA-sequencing approach revealed a bone marrow-derived macrophage subpopulation that drives tumor progression in the IDH1-wild-type glioblastomas [166]. The scavenger receptor MARCO expressed on the macrophages was associated with a worse prognosis and mesenchymal subtype of the tumor. Another potential target for immunotherapy, oncogene SPI1, was also identified in tumor-associated macrophages at a single-cell level [167]. SPI1 was essential for macrophage maturation and polarization, and its expression correlated with tumor grades and poor prognosis. Thus, single-cell RNA sequencing is a very useful tool for the analysis of intratumoral heterogeneity. It can be used to characterize both the TAAs profile of tumor cells subpopulations and the tumor cell microenvironment capable of promoting tumor growth and resistance to immunotherapy.

## 7. Conclusions

Gliomas are characterized by the inter-tumoral and intratumoral heterogeneity, which may be the reason for failures in the development of effective treatments. Genomic/transcriptomic/proteomic analysis of tumor tissue or bulk cell population provides the values of gene expression, the number of specific proteins and other indicators, averaged over all cells of the sample. As a result, information about minor subpopulations playing significant roles in oncogenesis and with the potential to be targets of prospective anti-cancer therapies is lost. At present, a number of high-throughput technologies for studying tumor tissue at the single-cell level, including flow cytometry, mass cytometry and RNA and DNA single-cell sequencing, are already widely used. The use of single-cell omics approach for studying cell heterogeneity has made it possible to obtain a more detailed characterization of the glioma cell composition. Single-cell sequencing of IDH-mutant gliomas, i.e., oligodendroglioma and astrocytoma, made it possible to see the hierarchy of glioma cell subpopulations. In this hierarchy, NPC-like cells give rise to both OC-like cells and AC-like cells. If so, targeted therapy triggering tumor cell differentiation towards astrocytes or oligodendrocytes would be potentially effective [168]. In case of IDH-wildtype glioblastomas, it was found that glioblastoma cells are represented by four transcriptional states, NPC-like, OPC-like, AC-like and MES-like. Importantly, proliferating cells are among all four cell subpopulations. Moreover, at the intersection of these lineages there is the fifth cell type, and its transcriptome is similar to that of GPCs. Moreover, it was shown that glioblastomas contain a subpopulation of radial glia-like cells which are probably at the top of the glioblastoma cell hierarchy and give rise to all other subtypes of glioblastoma cells. 

The revealed diversity of glioma stem cell subtypes may lead to a situation in which different groups of researchers isolate different cell states under the name “glioma CSCs”, and, as a result, observe the inconsistency of their data. Moreover, evidence has appeared that glioblastoma cells, under the microenvironment influence, can undergo reversible transitions between transcriptional states. If so, then the targets of therapy should be the signal pathways that mediate the plasticity of glioblastoma cells towards stem-like state [169]. Furthermore, tumor cell plasticity is an obstacle to the development of effective cell-based immunotherapy. To prevent tumor escape, an ideal immunotherapeutic effector cell should have multiple TAAs specificity capable of recognizing all possible variants resulting from cell heterogeneity. Obviously, the development of multicistronic CARs or similar approaches cannot solve this problem in full. Only an integrated therapeutic approach, including stroma-targeting antibodies, molecular inhibitors of cell plasticity and others, can be effective. Single-cell sequencing in combination with flow cytometry can provide almost complete information about potential molecular targets necessary to counter tumor cell heterogeneity.

It is also worth noting that in view of novel information about the difference in the cell composition of genetically different gliomas, future studies of glioma stem cells should be carried out taking into account the latest nosological classification. This will allow a more meaningful comparison of data from studies carried out by different researchers. Exploring the cell composition of glioma subtypes defined by molecular alterations may accelerate our understanding of glioma stem cell biology. Obviously, the different cell composition of tumors will require different targeted therapy. High hopes are also associated with the advances in single-cell multimodal omics technologies, since it will provide data on the relationship between phenotype, genotype, transcriptional state and epigenetic changes in each single cell of the tumor and specifically in putative CSCs.

## Figures and Tables

**Table 1:** Classification and key genetic mutations of the adult-type diffuse gliomas *.

Subtype of Adult-Type Diffuse Gliomas	Key Mutated Genes
Astrocytoma	IDH1, IDH2
Oligodendroglioma	IDH1, IDH2, 1p/19q-codeletion
Glioblastoma	IDH-wildtype

* Table is based on the summary of 2021 WHO classification of CNS tumors published by D.N. Louis et al. [5].

**Table 2:** Molecular markers of the glioma stem cells.

Cell Type	Intracellular Markers	Surface Markers
Glioma stem cells	Sox2, Nanog, Olig2, Myc, Musashi1, Bmi1, Nestin, Oct4, Brn2, ID1	CD133, CD15 (SSEA-1), α6 integrin, CD44, L1CAM, CD24, EGFR, PDGFRA, A2B5

**Table 3:** The capacities of current high-throughput single-cell technologies.

Method	Analyzed Entities	Number of Parameters Commonly Detected Per Cell	Number of Cells Commonly Analyzed in One Sample	Reference
Flow cytometry	Proteins and other antigens	Up to ten	Tens of thousands	[125]
Mass cytometry	Proteins and other antigens	Tens	Tens of thousands	[131]
Single-cell RNA sequencing	RNA molecules	Thousands	Thousands	[101]
Single-cell DNA sequencing	DNA regions (CNVs *, SNPs **, microsatellite mutations)	Thousands	Hundreds	[103]

* CNV—copy number variation; ** SNP—single nucleotide polymorphism.

## Data Availability

Not applicable.
